# Structures of the endophytic microbiota during heart rot development in *Abies georgei* var. *smithii*

**DOI:** 10.1128/spectrum.03904-25

**Published:** 2026-06-23

**Authors:** Yi Li, Yaxin Kong, Jieting Li, Chen Tang, Gengxin Zhang, Jiangrong Li, Yonglin Wang

**Affiliations:** 1 Key Laboratory of Forest Ecology in Tibet Plateau, Ministry of Education, Tibet Agricultural & Animal Husbandry University 667214 , Nyingchi, Tibet, China; 2 National Key Station of Field Scientific Observation & Experiment, Nyingchi, Tibet, China; 3 Beijing Key Laboratory for Forest Pest Control, College of Forestry, Beijing Forestry University 659739, Beijing, China; 4 Key Laboratory of Alpine Ecology and Biodiversity, Institute of Tibetan Plateau Research, Chinese Academy of Sciences 719567, Beijing, China; National Taiwan University, Taipei, Taiwan

**Keywords:** *Abies georgei *var. *smithii*, *Fomitopsis subpinicola*, heart rot, tree microbiome, bark microbiome, alpine ecosystem, high-throughput sequencing

## Abstract

Heart rot, caused by the basidiomycete fungus *Fomitopsis subpinicola*, poses a severe threat to the health of *Abies georgei* var. *smithii*, a keystone conifer dominating subalpine forests on Sejila Mountain in southeastern Xizang (Tibet), China. To understand the microbial dynamics associated with disease progression, we used 16S rRNA and internal transcribed spacer high-throughput sequencing combined with multivariate and co-occurrence network analyses to characterize structural changes in the trunk endophytic microbiota across healthy, asymptomatic (heartwood decay without external symptoms), and symptomatic (fruiting bodies present) trees. Heart rot progression is the dominant factor associated with microbial succession, explaining more variation than tissue compartment. The bark-associated microbiome exhibited the earliest and strongest shifts and may provide a useful target for future early assessment of heartwood decay. Microbial interaction networks, particularly cross-kingdom (bacteria-fungi) associations, exhibited a significant increase in negative correlations as disease progressed, suggesting a shift from predominantly positive or neutral associations toward more antagonistic interactions, which may reflect increasing ecological competition and progressive destabilization of the trunk microbiome during decay. A pivotal finding was the dynamic microbial response observed during the asymptomatic stage. At this stage, fungal communities had already diverged markedly, and disease-associated shifts involving taxa such as Vibrisseaceae and Microbacteriaceae suggested that microbial restructuring had begun before obvious external symptoms appeared.

**IMPORTANCE:**

Our findings show that shifts in endophytic microbiome structure and network stability are detectable during heart rot progression. In particular, bark-associated communities responded earlier and more strongly than near-pith communities, suggesting their potential value in future microbiome-informed, less destructive approaches for early assessment of cryptic stem diseases.

## INTRODUCTION

Alpine coniferous forests dominated by *Abies* spp. and other cold-tolerant conifers are widely distributed across the subalpine zones on the southeastern margin of the Qinghai–Tibet Plateau (1). These forests provide important ecological functions, including water conservation, soil retention, and biodiversity maintenance, and serve as a key ecological barrier in this region (2, 3). Among them, *Abies georgei* var. *smithii* (*A. georgei* var. *smithii*) is an endemic fir species that plays a foundational role in maintaining the structure and function of fragile high-altitude forest ecosystems. On the Sejila Mountain in southeastern Tibet, *A. georgei* var. *smithii* forms extensive natural dark coniferous forests. However, these forests are increasingly affected by severe heart rot, which reduces wood quality, weakens mechanical stability, and increases tree mortality, thereby causing substantial ecological and economic losses (4–6).

Recent field surveys and pathogenicity tests have shown that the dominant causal agent of heart rot in *A. georgei* var. *smithii* on Sejila Mountain is the basidiomycete fungus *Fomitopsis subpinicola* (7). This brown-rot fungus primarily degrades cellulose and hemicellulose in heartwood, leading to a progressive structural failure (8, 9). Infections are usually initiated through wounds or natural openings, after which the pathogen slowly colonizes the inner xylem over many years (9, 10). Because the disease develops cryptically, internal decay can expand substantially before obvious external symptoms appear. By the time visible signs such as fruiting bodies or external discoloration are observed, tree vigor, physiological functioning, and mechanical stability are often already severely compromised (6, 8, 11).

Wood decay is not simply the result of a single pathogenic fungus, but rather a complex ecological process involving multiple microbial groups and their interactions within the host (12). In primary dark coniferous forests on the southeastern Tibetan Plateau, previous studies have focused mainly on carbon storage and soil biogeochemical processes, whereas the dynamics of trunk-associated microbiota during heart rot development have received much less attention (13–16). During disease progression, changes in wood physicochemical properties are likely to alter the niches available to endophytic microorganisms. Studies in other forest systems have shown that endophytic and other wood-inhabiting microbiota respond sensitively to disease occurrence, and together with wood-decay fungi, contribute to wood decomposition and nutrient cycling (17–21). However, for high-altitude endemic conifers such as *A. georgei* var. *smithii*, the succession patterns of trunk-associated microbial communities during heart rot development remain poorly understood (9, 20).

Endophytic microorganisms in the xylem are important components of the internal tree microecosystem and may respond to decay progression before conspicuous morphological symptoms appear (22, 23). In healthy trees, these communities are generally more balanced, whereas disease progression may favor opportunistic colonizers and decomposer taxa adapted to changing substrate conditions (18, 22, 24, 25). This raises the possibility that microbiome restructuring may provide useful ecological insight into disease progression. At present, however, forest health assessment in *A. georgei* var. *smithii* still relies mainly on traditional approaches, particularly visual inspection of external symptoms and fruiting bodies. These methods have limited value for the early detection of cryptic heart rot because external symptoms appear late, and coring-based approaches are destructive.

Despite growing interest in the relationship between wood decay and microbial community dynamics, systematic microbial ecology studies of *A. georgei* var. *smithii* in fragile high-altitude forests remain scarce (6, 20). In particular, how trunk-associated bacterial and fungal communities differ among healthy, asymptomatic, and symptomatic trees, and how these patterns vary between the near-bark and near-pith compartments, remains poorly understood. To address these gaps, we investigated the trunk microbiota of *A. georgei* var. *smithii* on Sejila Mountain, southeastern Tibet, using 16S rRNA gene and internal transcribed spacer (ITS) amplicon sequencing combined with multivariate community analysis, differential abundance analysis, and co-occurrence network analysis.

Specifically, this study aimed to (i) characterize differences in bacterial and fungal community structure among healthy, asymptomatic, and symptomatic trees and between the near-bark and near-pith compartments; (ii) identify microbial taxa associated with these disease categories and tissue compartments; and (iii) assess whether fungal communities, bark-associated communities, or their network properties show stronger responses to disease progression than other community components. Together, these analyses provide an ecological framework for understanding trunk microbiome succession during heart rot development in *A. georgei* var. *smithii*.

## RESULTS

### Disease severity dominates microbial community structure variation

**Fig 1:**
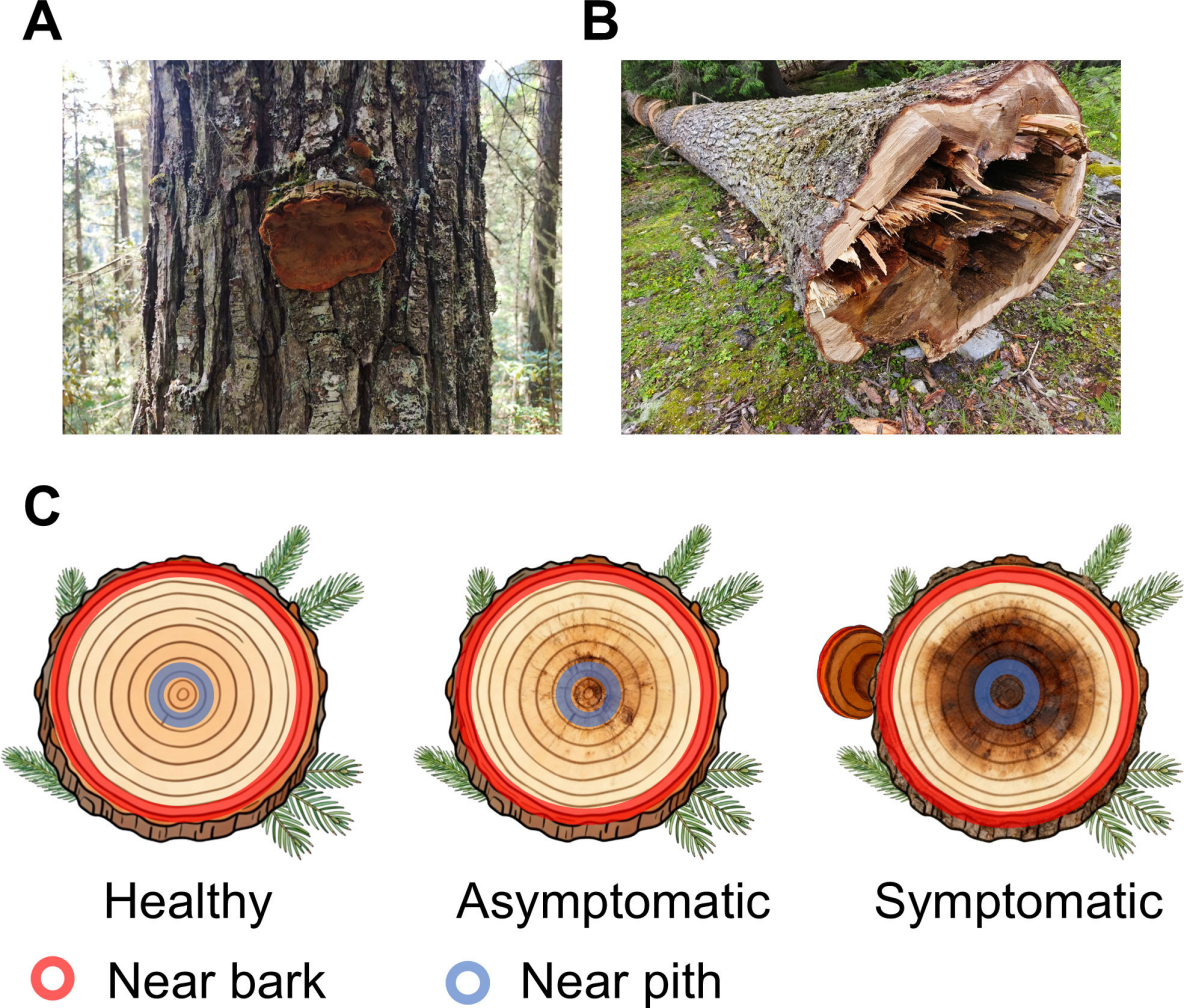
Representative symptoms of heart rot and schematic illustration of disease categories and sampling compartments. (**A**) Representative external symptom of symptomatic *Abies georgei* var. *smithii*, showing a basidiocarp on the trunk surface. (**B**) Representative internal symptom of heart rot, showing extensive hollow and decayed heartwood. (**C**) Schematic diagram of health-status grouping and sampling strategy used in this study. In panel C, Healthy, Asymptomatic, and Symptomatic indicate healthy trees, asymptomatic trees with internal decay but no obvious external symptoms, and symptomatic trees with fruiting bodies and severe internal decay, respectively. Near bark and Near pith indicate the two trunk positions sampled for microbiome analysis.

To investigate how heart rot is associated with changes in the trunk microbiome, we collected multisite samples from healthy, asymptomatic, and symptomatic *A. georgei* var. *smithii* trees across multiple sampling sites and analyzed their bacterial and fungal communities. Representative external and internal symptoms of heart rot, together with the disease classification and sampling scheme, are shown in Fig. 1. Tree health status was defined as follows: healthy (H), no external or internal abnormalities; asymptomatic (AS), normal bark surface but internal decay present; symptomatic (S), fruiting bodies present on the trunk surface, with advanced internal decay (Fig. 1; Table 1). To analyze within-trunk variation, we selected two tissue compartments from each increment core: an outer section located approximately 2–3 cm from the bark (near-bark compartment) and an inner section located approximately 2–3 cm from the pith (near-pith compartment). The bacterial and fungal communities were profiled by 16S rRNA gene and ITS amplicon sequencing, respectively. The overall effects of disease status and tissue compartment on microbial community structure, as assessed by PERMANOVA, are summarized in Table 2.

**TABLE 1:** Number of trunk core samples collected from healthy, asymptomatic, and symptomatic trees

	Near bark	Near pith	Total number of samples
Healthy	5	5	10
Asymptomatic	4	4	8
Symptomatic	5	5	10

**TABLE 2:** Decomposition of microbial community structure variation based on PERMANOVA^*a*^

Factor	df	SumsOfSqs	*R* ^2^	Pseudo-*F*	*P* value
Bacterial					
Disease	2	1.4	0.133	2.02	0.001
Compartment	1	0.61	0.058	1.76	0.018
Disease:compartment	2	0.86	0.081	1.23	0.105
Residuals	22	7.65	0.727	NA^*b*^	NA
Total	27	10.52	1	NA	NA
Fungal					
Disease	2	1.59	0.131	2	0.001
Compartment	1	0.65	0.054	1.64	0.015
Disease:compartment	2	1.15	0.095	1.45	0.014
Residuals	22	8.75	0.721	NA	NA
Total	27	12.14	1	NA	NA

^*a*^ The explanatory power (*R*^2^), pseudo-*F* values, and significance levels of disease status, tissue compartment, and their interaction on bacterial and fungal community structure variation are shown.

^*b*^ NA, not applicable.

**Fig 2:**
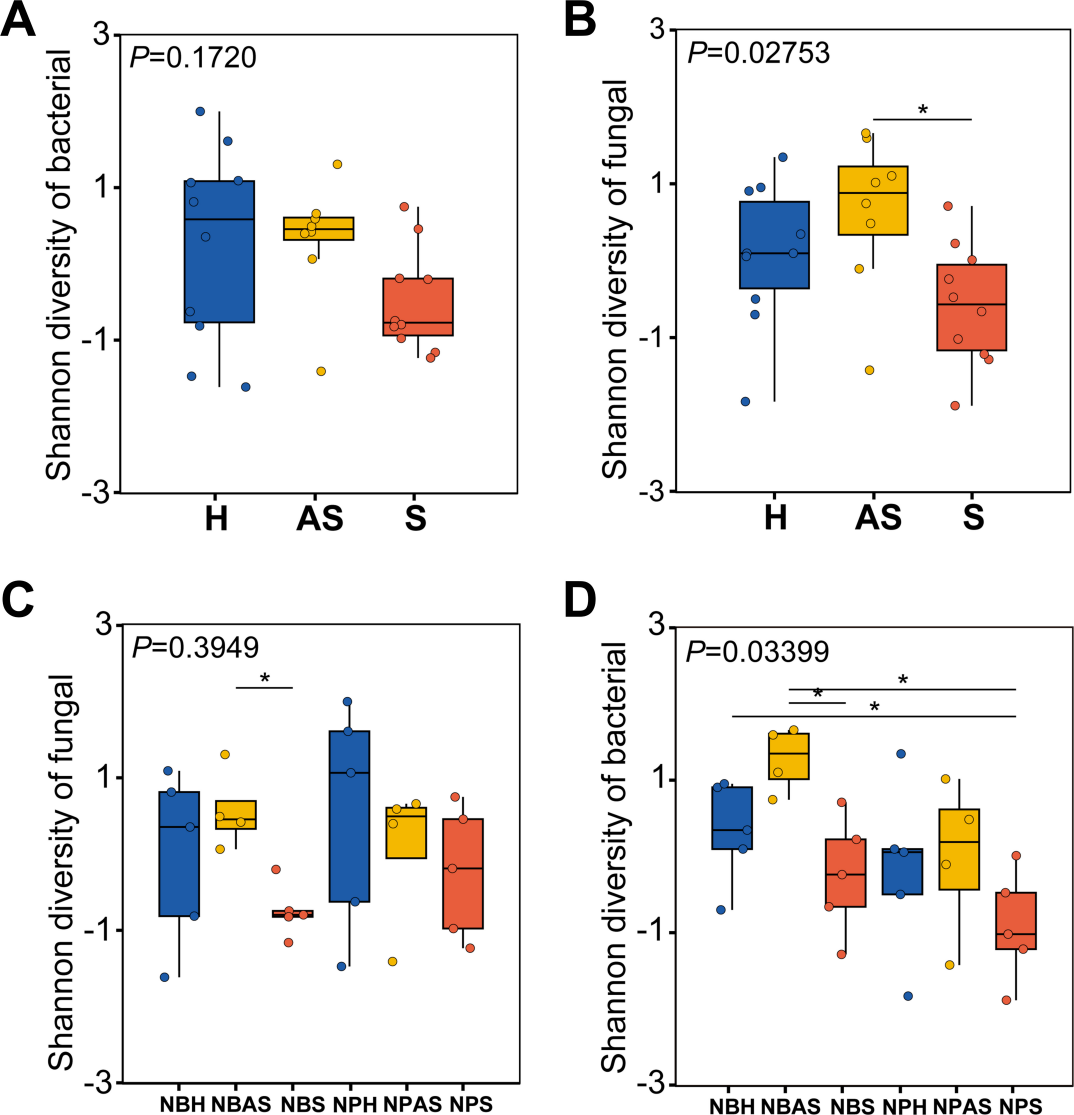
Alpha diversity (Shannon index) of bacterial and fungal communities. (**A**) Bacterial communities across health statuses; (**B**) fungal communities across health statuses; (**C**) bacterial communities by health status and tissue section; (**D**) fungal communities by health status and tissue section. Box plots depict the medians (central horizontal lines) and interquartile ranges (boxes). Sample groups: NBH, near-bark healthy trees; NBAS, near-bark asymptomatic trees; NBS, near-bark symptomatic trees; NPH, near-pith healthy trees; NPAS, near-pith asymptomatic trees; and NPS, near-pith symptomatic trees. Asterisks indicate significant differences (*P* < 0.05).

After quality control, host-sequence filtering, and denoising, the bacterial and fungal data sets were rarefied to 14,646 and 45,525 sequences per sample, respectively, for downstream diversity analyses. Bacterial Shannon diversity did not differ significantly among healthy, asymptomatic, and symptomatic samples (Fig. 2A). In contrast, fungal Shannon diversity differed significantly between asymptomatic and symptomatic samples (Fig. 2B). These alpha-diversity patterns suggest that disease development was associated with changes in overall fungal community diversity, whereas bacterial alpha diversity remained comparatively stable. However, because the Shannon index does not identify which taxa changed, these patterns should be interpreted together with community composition and network analyses. Further analysis of Shannon diversity by disease status and tissue compartment showed that significant differences between asymptomatic and symptomatic samples were detected only in the near-bark compartment for both bacterial and fungal communities (Fig. 2C and D). In contrast, bacterial and fungal communities in the near-pith compartment did not show significant pairwise differences among disease severity levels after multiple-testing correction (Tables 3 and 4). The magnitude of this compartment-specific difference was most evident in the asymptomatic-vs-symptomatic comparison, with significant separation in both bacterial (near-bark asymptomatic trees [NBAS] vs near-bark symptomatic trees [NBS], *P* = 0.007) and fungal (NBAS vs NBS, *P* = 0.005) communities at the near-bark compartment, whereas no corresponding significant differences were detected in the near-pith compartment. In addition, fungal communities in the near-bark compartment also showed a trend toward differentiation between healthy and asymptomatic trees. Taken together, these results indicate that the near-bark compartment captured the earliest and strongest disease-associated microbial shifts, particularly during the transition from asymptomatic to symptomatic stages.

**TABLE 3:** Pairwise comparisons of bacterial community structure with Bonferroni correction^*a*^

Comparison pairs	*R* ^2^	*P* value	Bonferroni-adjusted significance
H vs AS	0.087	0.040	No
H vs S	0.112	0.004	Yes
AS vs S	0.103	0.016	Yes
NPH vs NPAS	0.151	0.162	No
NPH vs NPS	0.168	0.016	No
NPAS vs NPS	0.152	0.102	No
NBH vs NBAS	0.141	0.190	No
NBH vs NBS	0.213	0.012	No
NBAS vs NBS	0.287	0.007	Yes

^*a*^ Significance was determined at *P* < 0.0167 for overall comparisons and *P* < 0.0083 for compartment-specific comparisons.

**TABLE 4:** Pairwise comparisons of fungal community structure with Bonferroni correction^*a*^

Comparison pairs	*R* ^2^	*P* value	Bonferroni-adjusted significance
H vs AS	0.105	0.005	Yes
H vs S	0.084	0.026	No
AS vs S	0.118	0.001	Yes
NPH vs NPAS	0.157	0.078	No
NPH vs NPS	0.149	0.197	No
NPAS vs NPS	0.152	0.238	No
NBH vs NBAS	0.274	0.013	No
NBH vs NBS	0.160	0.055	No
NBAS vs NBS	0.277	0.005	Yes

^*a*^ Significance was determined at *P* < 0.0167 for overall comparisons and *P* < 0.0083 for compartment-specific comparisons.

**Fig 3:**
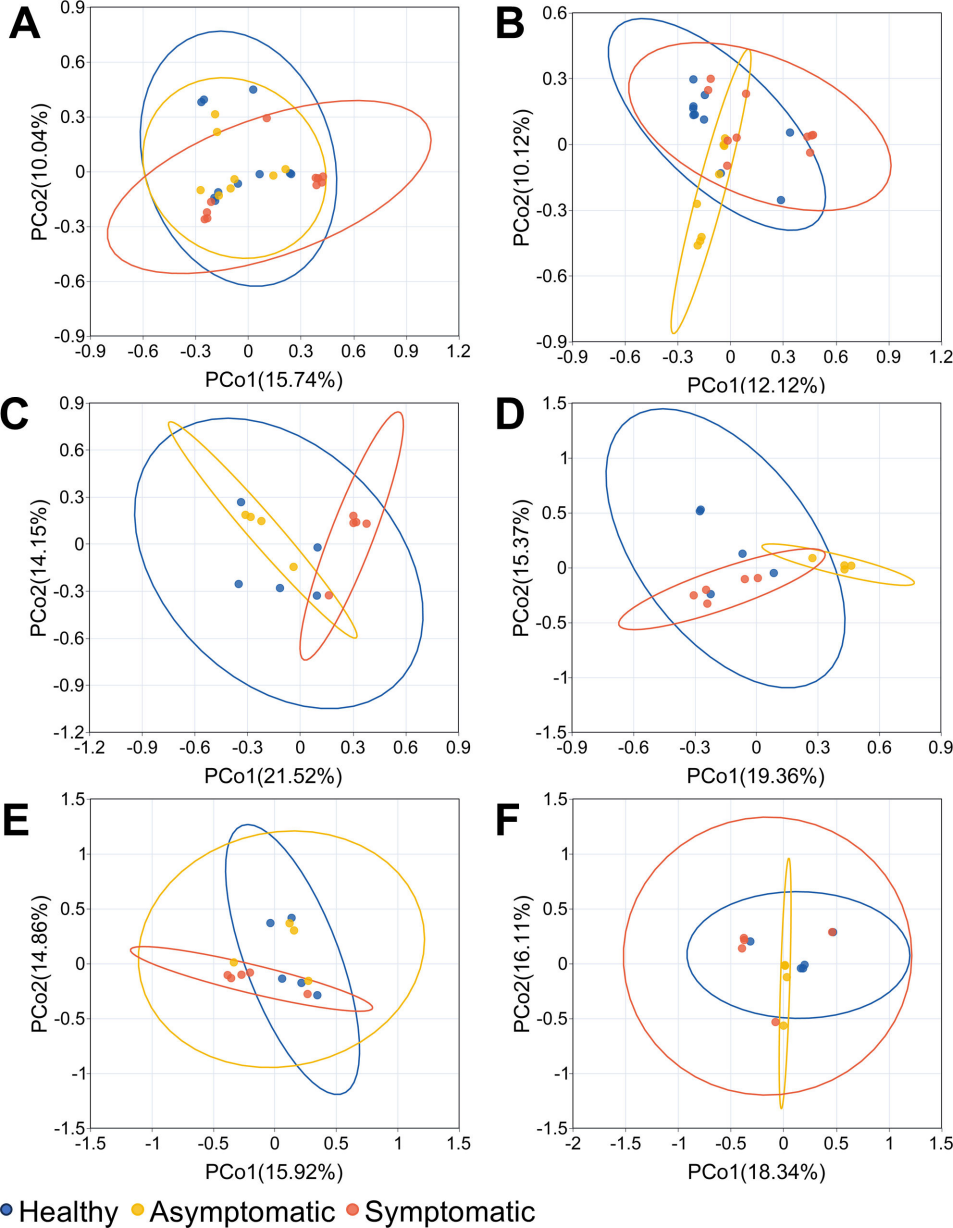
Principal coordinate analysis of bacterial and fungal communities based on Bray–Curtis distances. (**A**) Bacterial community structure across all samples. (**B**) Fungal community structure across all samples. (**C**) Bacterial community structure in the near-bark compartment. (**D**) Fungal community structure in the near-bark compartment. (**E**) Bacterial community structure in the near-pith compartment. (**F**) Fungal community structure in the near-pith compartment. Samples are colored by disease category: healthy, asymptomatic, and symptomatic. Ellipses represent 95% confidence intervals for each group. Overall PERMANOVA results are summarized in Table 2.

Principal coordinate analysis (PCoA) based on Bray–Curtis distances showed that bacterial communities from healthy and asymptomatic trees overlapped considerably, whereas symptomatic bacterial communities were more clearly separated (Fig. 3A). In contrast, fungal communities from asymptomatic trees were more distinct, whereas healthy and symptomatic fungal communities overlapped more strongly (Fig. 3B). Compartment-specific ordinations further indicated that disease-associated community differentiation was more pronounced in the near-bark compartment than in the near-pith compartment for both bacteria and fungi (Fig. 3C through F).

PERMANOVA indicated that disease status was the strongest factor associated with variation in both bacterial and fungal community structures, whereas tissue compartment explained a small but still significant proportion of the variation (Table 2). The interaction between disease status and tissue compartment was not significant for bacteria, but was significant for fungi (Table 2). In addition, a separate site-based PERMANOVA using the four sampling sites as grouping factors detected no significant site effect for either bacterial communities (Adonis, Bray–Curtis, *R* ^2^= 0.100, *P* = 0.769) or fungal communities (Adonis, Bray–Curtis, *R*^2^ = 0.094, *P* = 0.914), suggesting that sampling site was not a major source of variation in this data set. *Post hoc* pairwise comparisons (Bonferroni-corrected) further clarified these disease-associated patterns. For bacteria, the difference between healthy and asymptomatic trees was not significant, whereas healthy vs symptomatic and asymptomatic vs symptomatic trees were significantly different (Table 3). For fungi, healthy vs symptomatic trees did not differ significantly, whereas healthy vs asymptomatic and asymptomatic vs symptomatic trees were significantly different (Table 4).

To further examine compartment-specific effects across disease stages, we compared the microbiomes among disease groups separately within each tissue compartment. For bacteria, after Bonferroni correction, a significant difference between asymptomatic and symptomatic samples was detected only in the near-bark compartment (*P* = 0.007). In the healthy-vs-symptomatic comparison, similar patterns were observed in both the near-bark and near-pith compartments at the nominal significance level, although these pairwise differences did not remain significant after Bonferroni correction (Table 3). Similarly, for fungi, a significant difference between asymptomatic and symptomatic samples was detected only in the near-bark compartment (*P* = 0.005). A nominally significant trend was also observed for the healthy-vs-asymptomatic comparison in the near-bark compartment, but this difference did not remain significant after Bonferroni correction (Table 4). Overall, these comparisons indicate that disease-associated microbial divergence was concentrated in the near-bark compartment, particularly during the transition from asymptomatic to symptomatic stages.

### Overall composition dynamics at the class and family levels

To systematically reveal the specific impact of heart rot on the structure and dynamics of the tree microbiome, we first conducted a comprehensive analysis of the overall compositional changes at both class and family levels. This foundational analysis was followed by rigorous statistical validation to identify key taxonomic groups that exhibited significant shifts in abundance across the disease continuum.

**Fig 4:**
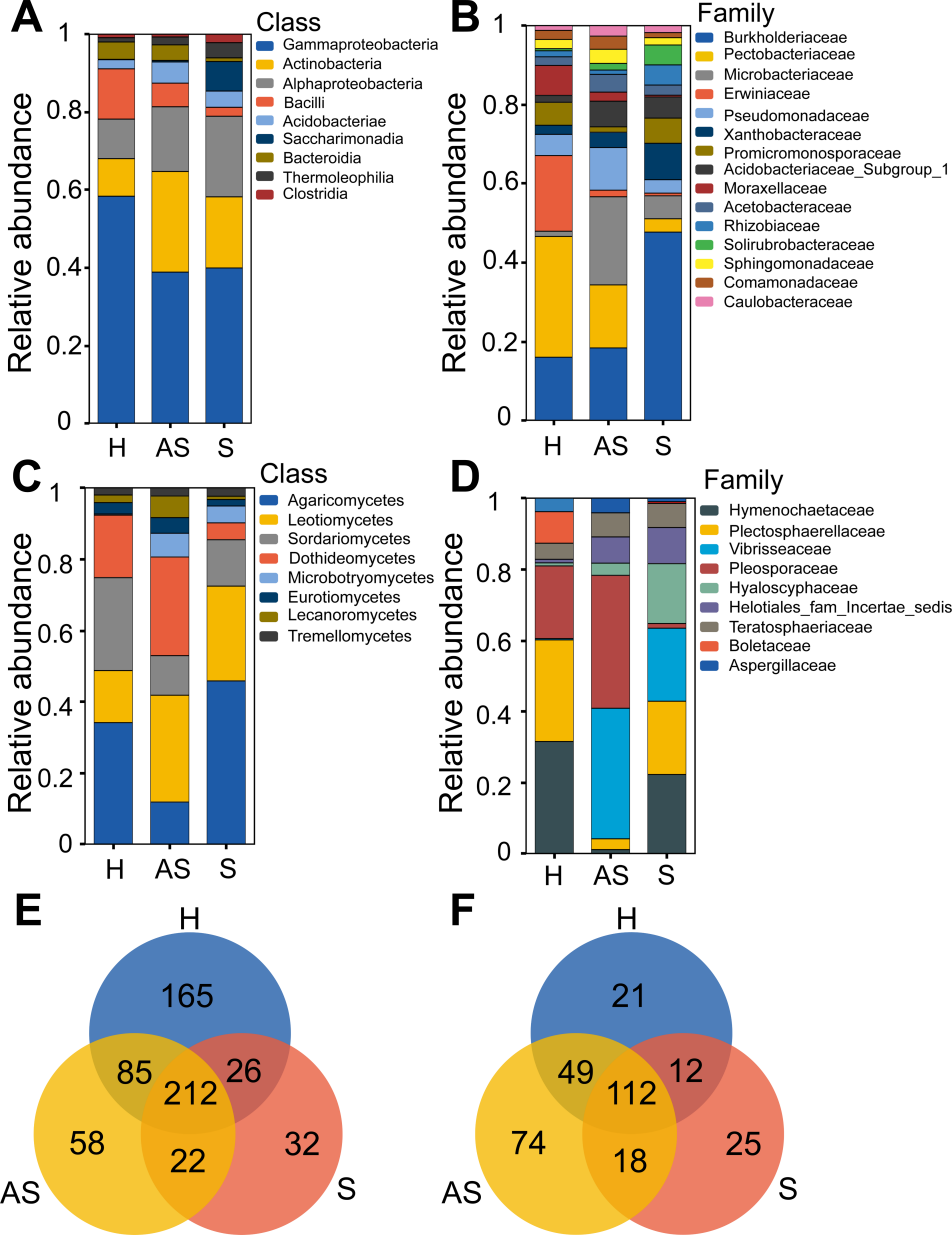
Analysis of microbial community composition. (**A**) Composition of the bacterial community at the class level; (**B**) composition of the bacterial community at the family level; (**C**) composition of the fungal community at the class level; (**D**) composition of the fungal community at the family level; (**E**) Venn diagram of bacterial genera; (**F**) Venn diagram of fungal genera.

Our bacterial community analysis identified 52 phyla, 128 classes, 262 orders, 332 families, and 565 genera. Initial examination at the class level revealed that the community was predominantly composed of Gammaproteobacteria and Alphaproteobacteria within the phylum Proteobacteria, with substantial contributions from Actinobacteria and Bacilli (Fig. 4A). A marked succession was observed as the disease state advanced; the initially dominant Gammaproteobacteria, which constituted 58.3% of the community in healthy trees, experienced a considerable decline in abundance during the disease stages. Conversely, Actinobacteria increased markedly, with its mean relative abundance reaching 25.9% in the asymptomatic group, compared with lower average levels in the healthy and symptomatic groups. This microbial succession pattern was even more pronounced and detailed at a higher resolution at the family level (Fig. 4B). Parallel analysis of the fungal community revealed 7 phyla, 24 classes, 73 orders, 183 families, and 310 genera. At the class level, the fungal community in the healthy group was dominated by Agaricomycetes (34.1%) and Sordariomycetes (26.1%). In contrast, the asymptomatic stage presented a distinct profile characterized by a notable dominance of Leotiomycetes (30.0%) and Dothideomycetes (27.7%) (Fig. 4C; Table S1).

In addition, at the family level within the fungal community, Vibrisseaceae (Leotiomycetes) emerged as a key player, and its abundance increased dramatically to 36.7% in the asymptomatic group, establishing it as a hallmark taxon for this specific disease stage (Fig. 4D; Table S1). In contrast, Hymenochaetaceae (Agaricomycetes) maintained a relatively high and stable abundance across both healthy and symptomatic groups (Fig. 4D; Table S1).

To complement the compositional analyses, Venn diagrams were used to delineate shared and unique microbial genera across different health states. In the bacterial community, the healthy, asymptomatic, and symptomatic groups shared a core of 212 genera. The number of genera unique to the healthy, asymptomatic, and symptomatic groups was 165, 58, and 32, respectively (Fig. 4E). Analysis of the fungal communities revealed that the three groups shared 112 genera. The number of unique fungal genera in the healthy, asymptomatic, and symptomatic groups was 21, 74, and 25, respectively (Fig. 4F). Full differential abundance results for fungal taxa at the phylum, class, and genus levels are provided in Table S2. Full differential abundance results for bacterial taxa at the phylum, class, and genus levels are provided in Table S3. To better visualize within-group variation across individual samples, sample-level bacterial and fungal family composition profiles are provided in Fig. S1.

### Pairwise difference analysis identifies taxa associated with disease stages

**Fig 5:**
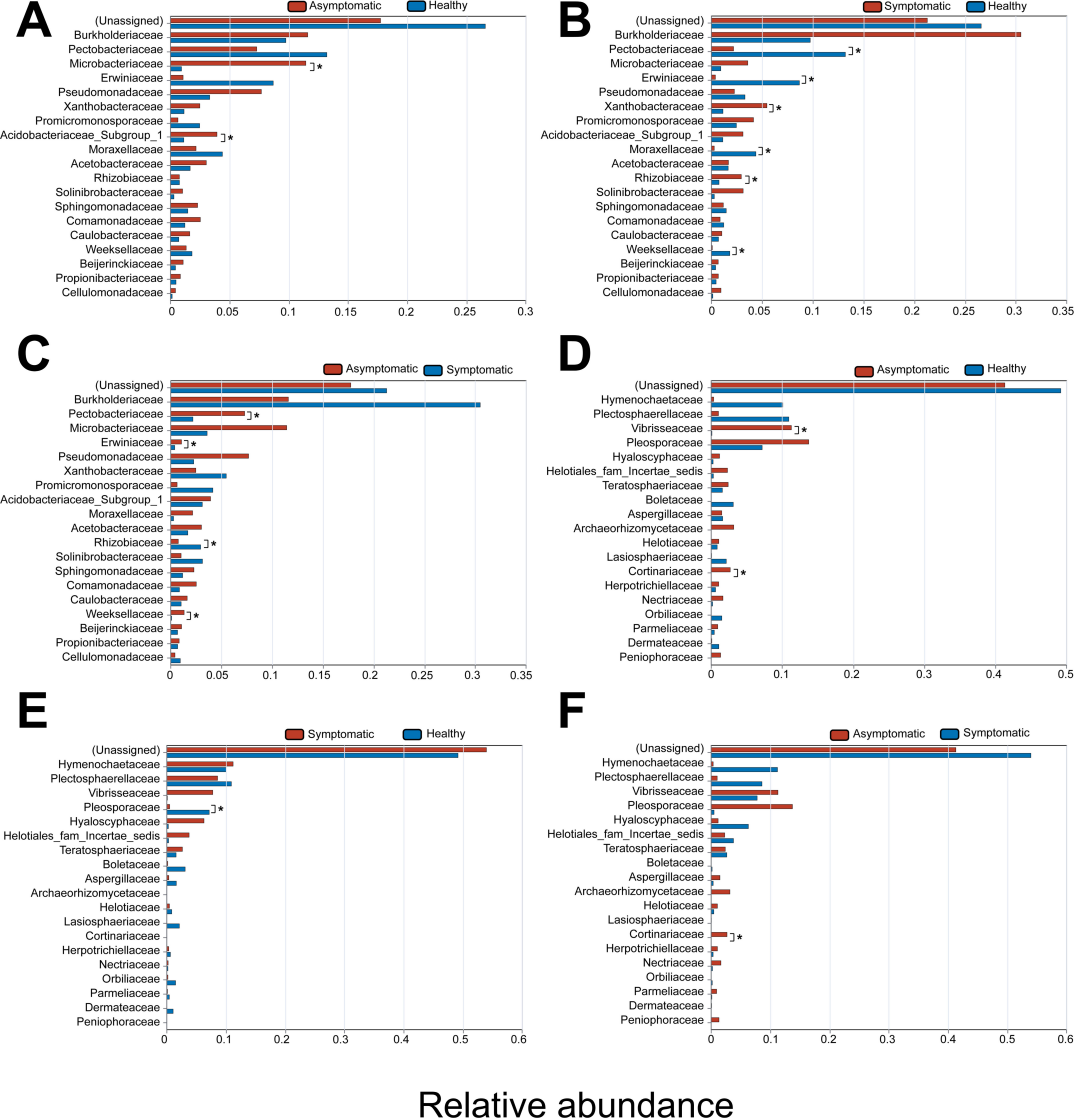
Pairwise comparisons of the top 20 most abundant bacterial and fungal families among tree health statuses. (**A–C**) Significantly differentially abundant bacterial families in (**A**) H vs AS, (**B**) H vs S, and (**C**) AS vs S. (**D–F**) Significantly differentially abundant fungal families in (**D**) H vs AS, (**E**) H vs S, and (**F**) AS vs S. Significance was determined using the Wilcoxon rank-sum test (FDR-adjusted *P* < 0.05). Only taxa meeting the significance threshold are shown. *P < 0.05*.*

The observed compositional shifts were further validated by pairwise difference analysis, which identified specific bacterial and fungal families whose abundances were significantly associated with different disease stages. Several families exhibited significant responses within the bacterial community. The relative abundance of Microbacteriaceae (Actinobacteriota) increased significantly from 1.3% in the healthy group to 22.3% in the asymptomatic group, indicating a significant difference between these two groups (Fig. 5A). In contrast, Pectobacteriaceae and Erwiniaceae, which were more abundant in healthy trees, were significantly depleted in symptomatic trees relative to both healthy and asymptomatic trees (Fig. 5B and C). Although Burkholderiaceae became the most dominant family in the symptomatic group (47.7%), its overall variation across the three groups did not reach the preset significance threshold in the multi-group comparison (Table S4). However, Xanthobacteraceae and Rhizobiaceae were significantly different between the healthy and symptomatic groups (Fig. 5B).

Pairwise difference analysis also confirmed the role of specific taxa within the fungal community. Vibrisseaceae and Cortinariaceae were the key families whose abundances differed significantly between the healthy and asymptomatic groups (Fig. 5D). The dynamics of Pleosporaceae were also clarified: its abundance increased significantly in the asymptomatic group relative to the healthy group, and although it continued to increase in the symptomatic group, the difference between the asymptomatic and symptomatic groups was not statistically significant (Fig. 5E and F). Finally, the analysis confirmed that Hymenochaetaceae, despite its persistent presence, did not differ significantly among groups (Fig. 5E).

### Microbial co-occurrence networks reveal the disease impact on community stability

**Fig 6:**
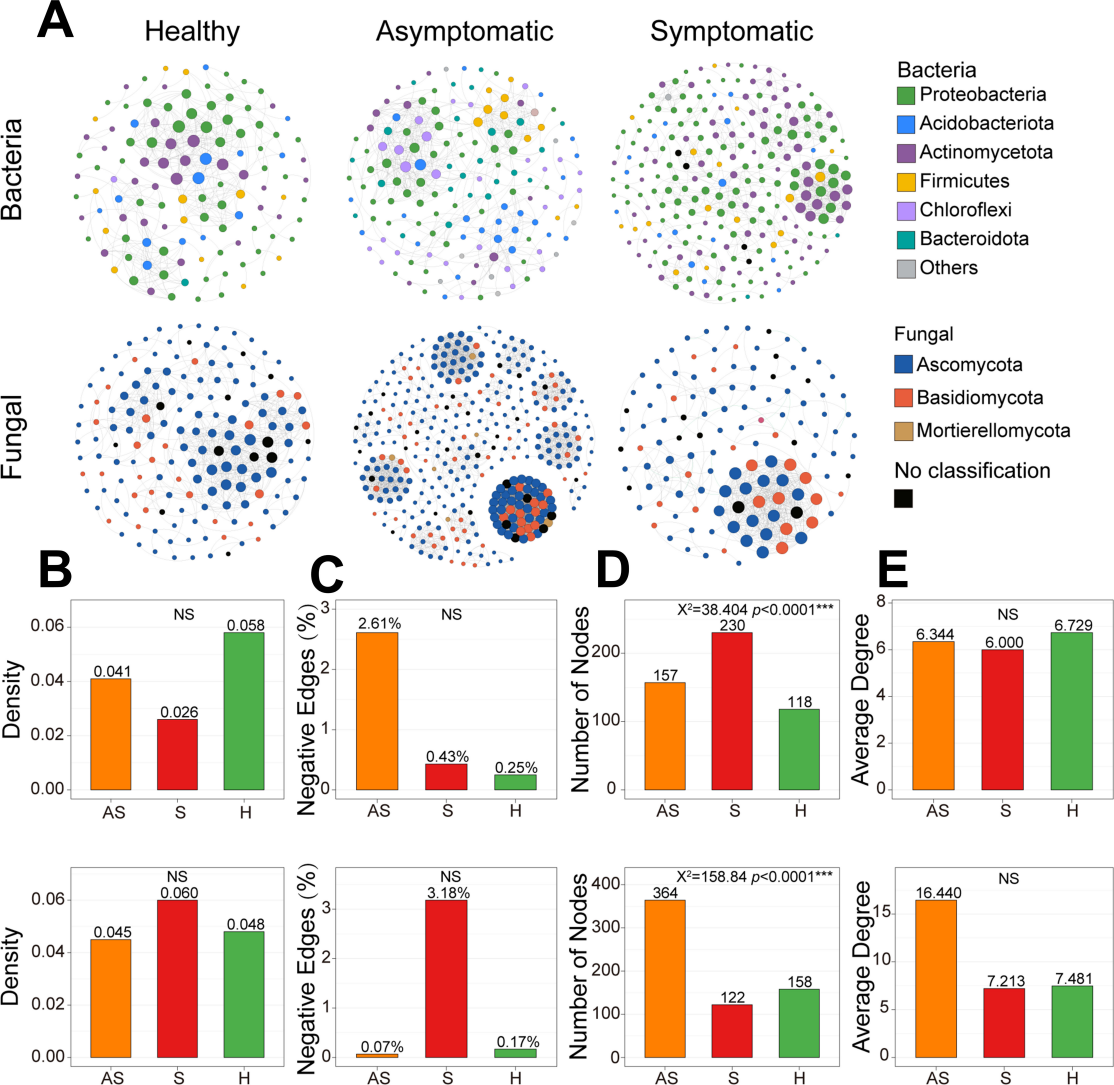
Intrakingdom microbial co-occurrence networks and their topological properties. (**A**) Bacterial and fungal co-occurrence network; (**B**) network density (top, bacteria; bottom, fungi); (**C**) proportion of negative edges (top, bacteria; bottom, fungi); (**D**) number of nodes (top, bacteria; bottom, fungi); (**E**) average degree (top, bacteria; bottom, fungi).

To elucidate the effects of heart rot on microbial interaction patterns, we constructed intrakingdom (bacteria-bacteria and fungal-fungal) and cross-kingdom (bacterial-fungal) co-occurrence networks for the healthy, asymptomatic, and symptomatic groups. Analysis of intrakingdom network topological parameters indicated disease-associated reorganization of microbial interaction patterns, with significant differences detected primarily in node numbers rather than in network density, the proportion of negative edges, or average degree (Tables 5 and 6). In bacterial intrakingdom networks (Fig. 6A), network density decreased progressively from 0.058 in the healthy group to 0.041 in the asymptomatic group and further to 0.026 in the symptomatic group, although these differences were not statistically significant (Fig. 6B; Table 5). In contrast, fungal intrakingdom network density showed only limited variation across disease stages, changing from 0.048 in the healthy group to 0.045 in the asymptomatic group and 0.060 in the symptomatic group, with no significant intergroup differences (Fig. 6B; Table 6). The proportion of negative correlations also varied across disease stages but did not differ significantly among groups. In bacterial networks, the proportion of negative correlations was highest in the asymptomatic group (2.61%), compared with 0.25% in the healthy group and 0.43% in the symptomatic group. In fungal networks, the proportion of negative correlations increased markedly in the symptomatic group to 3.18%, compared with 0.17% in the healthy group and 0.07% in the asymptomatic group (Fig. 6C). Node numbers changed markedly across disease stages: bacterial node counts increased from 118 in the healthy group to 157 in the asymptomatic group and 230 in the symptomatic group (*χ*² = 38.404, *P* < 0.0001), whereas fungal node counts increased from 158 in the healthy group to 364 in the asymptomatic group before declining to 122 in the symptomatic group (*χ*² = 158.84, *P* < 0.0001) (Fig. 6D). Average degree did not differ significantly among groups. In bacterial networks, the average degree declined slightly from 6.729 in the healthy group to 6.344 in the asymptomatic group and 6.000 in the symptomatic group, whereas in fungal networks it peaked in the asymptomatic group (16.440) compared with the healthy (7.481) and symptomatic (7.213) groups (Fig. 6E). Overall, these intrakingdom patterns suggest that fungal interactions were substantially reorganized during disease progression, with the asymptomatic stage representing a transitional state characterized by elevated network complexity before simplification in symptomatic trees.

**TABLE 5:** Statistics of topological parameters for intrakingdom (bacteria-bacteria) co-occurrence networks

Disease status	Total nodes	Total edges	Modularity	Network density	Average degree
Healthy	118	397	0.593	0.058	6.729
Asymptomatic	157	498	0.641	0.041	6.344
Symptomatic	230	690	0.818	0.026	6.000

**TABLE 6:** Statistics of topological parameters for intrakingdom (fungal-fungal) co-occurrence networks

Disease status	Total nodes	Total edges	Modularity	Network density	Average degree
Healthy	158	591	0.641	0.048	7.481
Asymptomatic	364	2,992	0.752	0.045	16.440
Symptomatic	122	440	0.437	0.060	7.213

**Fig 7:**
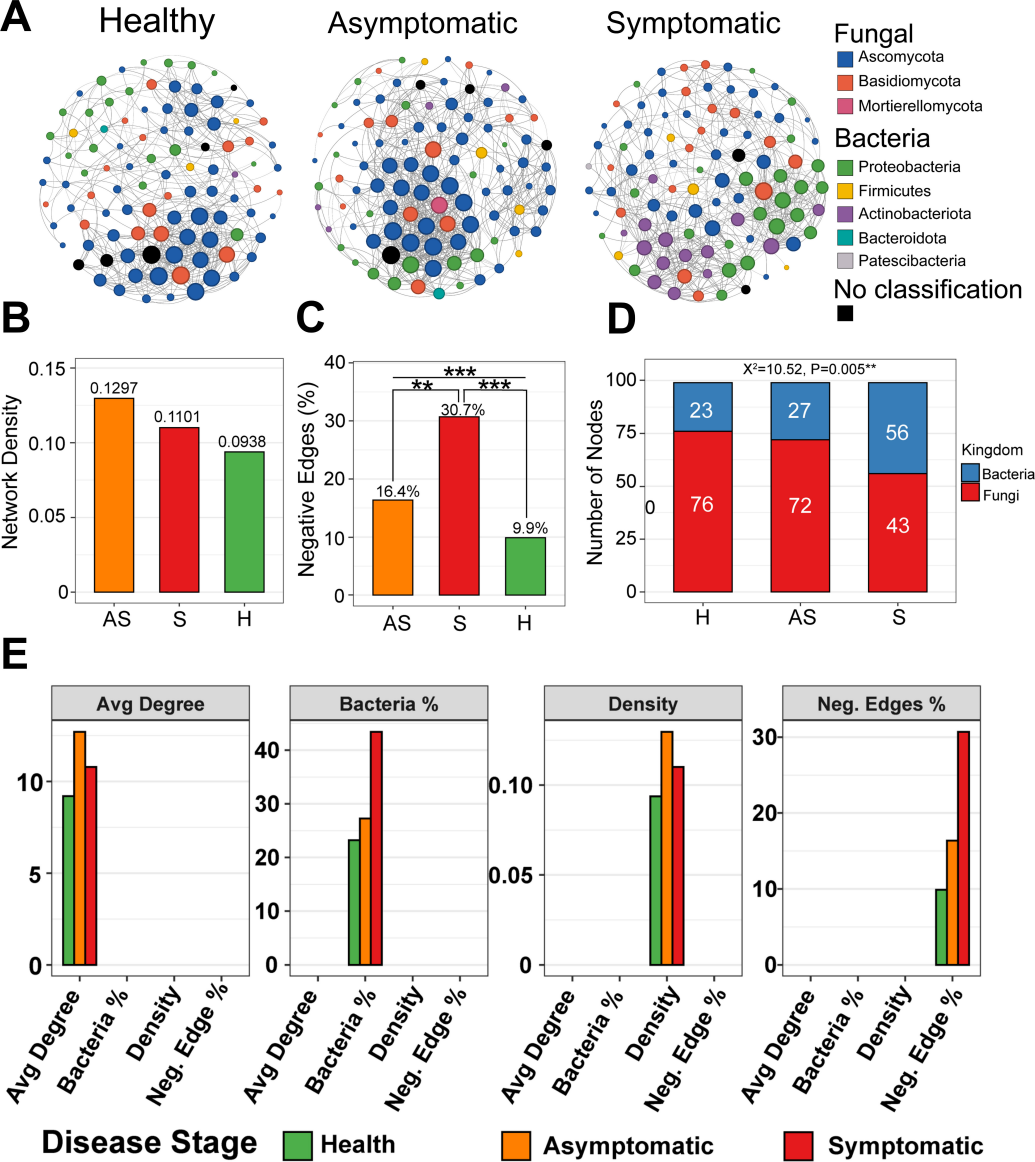
Cross-kingdom (bacteria-fungi) co-occurrence network analysis. (**A**) Network visualization; (**B**) changes in network density; (**C**) changes in the proportion of positive and negative edges; (**D**) changes in the distribution of bacterial and fungal nodes; (**E**) comprehensive comparison of key network parameters.

To gain a more comprehensive understanding of overall microbial interaction dynamics, we further analyzed cross-kingdom interactions between bacteria and fungi. The cross-kingdom co-occurrence networks showed clear topological shifts across the healthy, asymptomatic, and symptomatic groups (Fig. 7A). Network density followed a biphasic pattern, increasing from 0.0938 in the healthy group to 0.1297 in the asymptomatic group and then decreasing to 0.1101 in the symptomatic group; however, these intergroup differences were not statistically significant (Fig. 7B; Table 7). In contrast, the proportion of negative edges increased significantly with disease progression, rising from 9.9% in the healthy group to 16.4% in the asymptomatic group (*χ*² = 8.88, *P* = 0.003) and further to 30.7% in the symptomatic group (vs healthy: *χ*² = 62.66, *P* < 0.001; vs asymptomatic: *χ*² = 32.76, *P* < 0.001) (Fig. 7C). Correspondingly, the proportion of positive bacterial–fungal associations declined from 90.1% in the healthy network to 69.3% in the symptomatic network (Fig. 7C; Table 7).

**TABLE 7:** Statistics of topological parameters for cross-kingdom (bacteria-fungal) co-occurrence networks

Disease status	Total nodes	Total edges	Bacteria nodes	Fungi nodes	Positive edges	Negative edges	Network density	Ratio of bacteria/fungi nodes
Healthy	99	455	23	76	410	45	0.094	23/76
Asymptomatic	99	629	27	72	526	103	0.130	27/72
Symptomatic	99	534	43	56	370	164	0.110	43/56

The kingdom distribution of network nodes also changed significantly across disease stages (*χ*² = 10.52, *P* = 0.005). Specifically, bacterial node counts increased from 23 in the healthy group to 27 in the asymptomatic group and 56 in the symptomatic group, whereas fungal node counts decreased from 76 in the healthy group to 72 in the asymptomatic group and 43 in the symptomatic group (Fig. 7D), indicating an increased relative representation of bacteria in the late-stage cross-kingdom interaction network.

A comparative summary of four network parameters—average degree, bacterial proportion, network density, and the proportion of negative correlations—showed that negative correlations underwent the most pronounced change during disease progression (Fig. 7E). In addition, the numbers of nodes belonging to major phyla also changed dynamically across disease stages (Table 8). For example, Actinobacteriota-associated nodes increased markedly from 1 in the healthy network to 14 in the symptomatic network, whereas Ascomycota-associated nodes decreased from 53 to 38. In contrast, the number of Proteobacteria- and Basidiomycota-associated nodes varied relatively little among groups.

**TABLE 8:** Distribution of node numbers for key phylum-level taxa in cross-kingdom co-occurrence networks^*a*^

	Healthy	Asymptomatic	Symptomatic
Ascomycota	53	54	38
Proteobacteria	18	18	24
Actinobacteriota	1	4	14
Basidiomycota	18	13	16

^*a*^ The changes in node numbers of major bacterial phyla (Proteobacteria and Actinobacteria) and fungal phyla (Ascomycota and Basidiomycota) across different disease stages are shown.

### Identification of disease-associated microbes

Linear discriminant analysis (LDA) effect size (LEfSe) was used to identify taxa that differed significantly in abundance among disease severity groups, using an LDA score threshold of >3.5 and *P* < 0.05. The bar plots show representative discriminatory taxa across groups, with blue indicating healthy, green indicating asymptomatic, and red indicating symptomatic samples.

**Fig 8:**
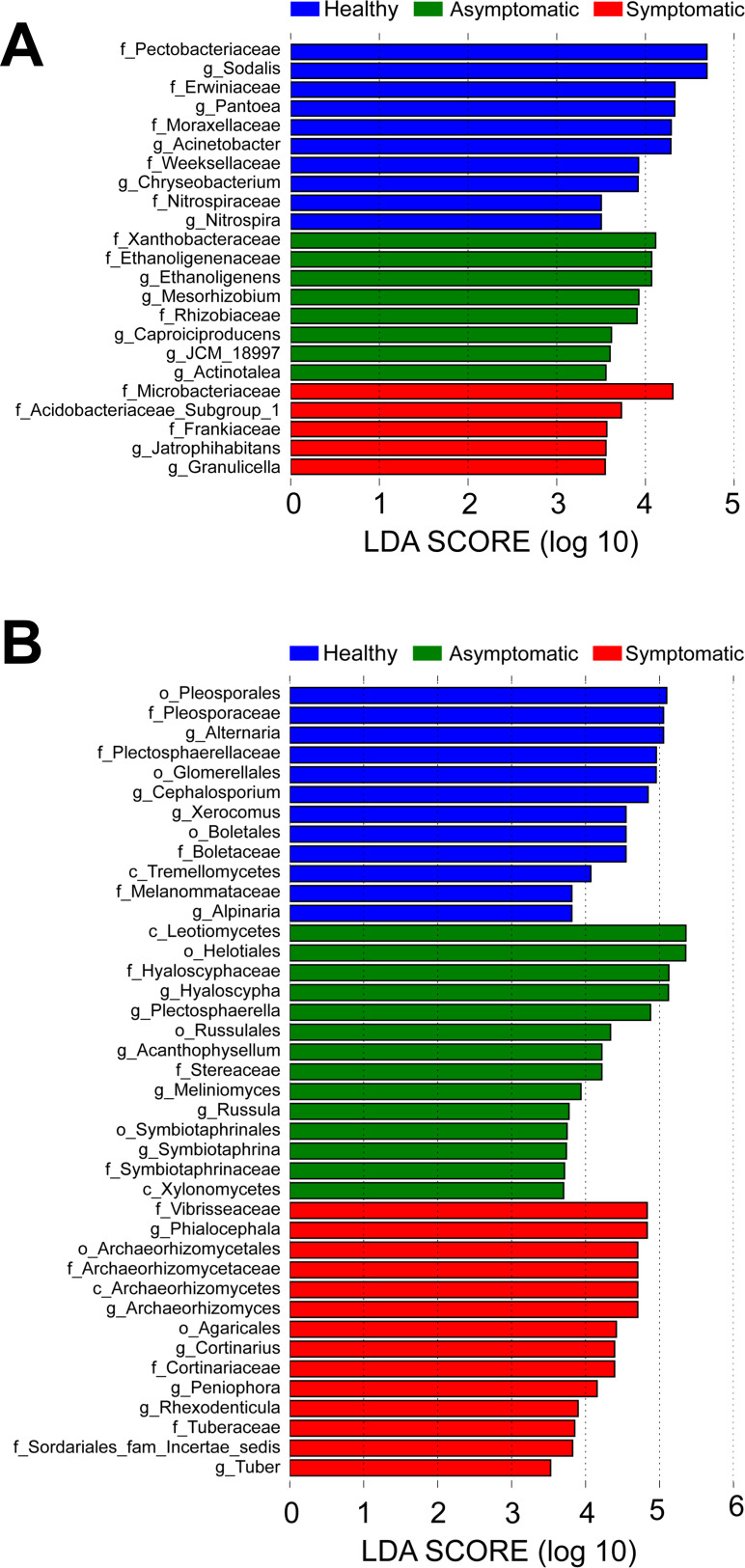
Disease-associated microbial biomarkers identified by LEfSe analysis. (**A**) Bacterial biomarkers. (**B**) Fungal biomarkers. LEfSe was used to identify discriminatory taxa across multiple taxonomic ranks among healthy, asymptomatic, and symptomatic samples, with health status as the class. Only taxa meeting the thresholds of Kruskal–Wallis *P* < 0.05 and LDA score >3.5 are shown.

At the bacterial level (Fig. 8A), the healthy group was characterized by enrichment of taxa such as Pectobacteriaceae, Erwiniaceae, Moraxellaceae, Weeksellaceae, and Nitrospiraceae, as well as the genera *Sodalis*, *Pantoea*, *Acinetobacter*, *Chryseobacterium*, and *Nitrospira*. The asymptomatic group was distinguished by enrichment of Xanthobacteraceae, Ethanoligenenaceae, and Rhizobiaceae, together with the genera *Ethanoligenens*, *Mesorhizobium*, *Caproiciproducens*, *JCM_18997*, and *Actinotalea*. In contrast, the symptomatic group was characterized by enrichment of Microbacteriaceae, Acidobacteriaceae_Subgroup_1, and Frankiaceae, as well as the genera *Jatrophihabitans* and *Granulicella*.

At the fungal level (Fig. 8B), the healthy group showed enrichment of taxa, including Pleosporaceae, Plectosphaerellaceae, Boletaceae, and Melanommataceae, together with the genera *Alternaria*, *Cephalosporium*, and *Xerocomus*. The asymptomatic group was characterized by enrichment of Hyaloscyphaceae, Stereaceae, and Symbiotaphrinaceae, as well as the genera *Hyaloscypha*, *Plectosphaerella*, *Acanthophysellum*, *Meliniomyces*, *Russula*, and *Symbiotaphrina*. In contrast, the symptomatic group was distinguished by enrichment of Vibrisseaceae, Archaeorhizomycetaceae, Cortinariaceae, and Tuberaceae, together with the genera *Phialocephala*, *Archaeorhizomyces*, *Cortinarius*, *Peniophora*, *Rhexodenticula*, and *Tuber*.

## DISCUSSION

Our results showed that disease status was more strongly associated with endophytic microbiome restructuring in *A. georgei* var. *smithii* than tissue compartment. For both bacteria and fungi, disease status explained a larger proportion of community variation than tissue compartment, indicating that decay progression acts as a strong ecological filter reshaping the trunk microbiome. Consistent with this interpretation, a separate site-based PERMANOVA detected no significant effect of sampling site on either bacterial or fungal community structure. Similar disease-associated restructuring has been reported in other wood decay and heart rot systems, including Heterobasidion-induced wood decay in Norway spruce and heart rot progression in living conifers (20, 26). Comparable stage-dependent microbiome shifts have also been documented in soil-borne wilt diseases of trees, such as Verticillium wilt of smoke trees (20, 24, 27). At the same time, the tissue compartment still contributed to community differentiation. The near-bark and near-pith compartments likely represent distinct microenvironments within the trunk, differing in oxygen availability, moisture conditions, host-derived compounds, and proximity to active transport tissues (18). Such within-trunk heterogeneity may help explain why bark-associated communities responded more strongly to disease progression than communities near the pith.

A major finding of this study was that the asymptomatic stage represented a critical transition, particularly for the fungal community. At this stage, fungal communities were more distinct from both healthy and symptomatic trees, whereas bacterial communities remained comparatively similar to those of healthy trees and shifted more markedly only at the symptomatic stage. LEfSe analysis further highlighted stage-associated fungal biomarkers: healthy trees were associated with Pleosporaceae, Plectosphaerellaceae, and Boletaceae, asymptomatic trees were enriched in Hyaloscyphaceae, Stereaceae, and Symbiotaphrinaceae, whereas symptomatic trees were characterized by Vibrisseaceae, Archaeorhizomycetaceae, Cortinariaceae, and Tuberaceae. This apparent decoupling between fungal and bacterial responses suggests that fungi may respond earlier during heart rot development, whereas bacteria become more prominent only after the substrate has been substantially modified. Similar successional sequences, in which fungal colonization and wood modification precede greater bacterial involvement, have also been reported in other wood-decomposition systems and woody tissues (17, 24, 25).

The taxonomic shifts observed here provide additional ecological clues. Pairwise family-level analysis showed that the relative abundance of Microbacteriaceae increased significantly from healthy to asymptomatic trees, suggesting that bacteria with potential antagonistic or protective functions may become more prominent during the early phases of fungal invasion. Several *Microbacterium* strains, which belong to the family Microbacteriaceae, have been reported to suppress plant-pathogenic fungi and enhance host defense responses in other systems (28). During the asymptomatic stage, the enrichment of taxa associated with oxygen-limited or fermentative conditions, especially Ethanoligenenaceae and *Ethanoligenens*, may indicate an increasingly reduced and nutrient-rich microenvironment accompanying early decay development (9, 24). Notably, different analytical frameworks highlighted partially different disease-associated taxa. Family-level composition and pairwise comparisons suggested that changes in some taxa became apparent already during the asymptomatic stage, whereas LEfSe identified some taxa as stronger discriminatory features of symptomatic samples. This difference likely reflects the distinct statistical emphases of abundance-based pairwise comparisons vs multigroup discriminant analysis rather than a true biological contradiction. Together, these patterns suggest a plausible successional trajectory from health to cryptic infection and then to overt decay.

Network analyses further indicated that disease progression was accompanied by a marked increase in negative correlations within cross-kingdom networks and by a higher proportion of bacterial nodes. The increase in negative edges suggests intensifying competition and declining community cohesion as decay advances. This pattern is broadly consistent with the “Anna Karenina principle” for microbiomes, under which stressed or diseased microbial communities tend to become less stable and more variable than healthy ones (22). In our system, this instability was reflected not only in taxonomic turnover but also in a shift from predominantly positive or neutral associations toward more antagonistic configurations, especially at later stages of disease (25, 29).

Another important result was the heightened sensitivity of the bark-associated microbiome. Significant differences in both bacterial and fungal communities between asymptomatic and symptomatic trees were detected only in the near-bark compartment, whereas the near-pith communities were comparatively conservative. This suggests that microbes integrate systemic signals of internal decay earlier than the inner heartwood community, possibly through their exposure to host defense compounds, sap flow, and microenvironmental changes along the stem. From an applied perspective, this finding suggests that bark sampling may be useful in future development of less destructive approaches for the early assessment of cryptic heart rot, complementing or partially replacing invasive coring-based diagnostics (30, 31). In this context, alpha-diversity metrics such as the Shannon index are unlikely to serve as stand-alone indicators, but they may still be informative when interpreted together with compartment-specific shifts in beta diversity, differential taxa, and network structure.

This study had several limitations. First, amplicon sequencing provides high-resolution taxonomic profiles but cannot directly resolve functional activities or interaction mechanisms; thus, the ecological roles of enriched taxa such as Microbacteriaceae and Vibrisseaceae remain to be experimentally verified (22, 26). Second, co-occurrence networks are based on statistical correlations and do not necessarily represent direct ecological interactions. Third, the number of trees in each health category was modest, and although site-based PERMANOVA did not detect a significant effect of sampling site, we did not explicitly model other fine-scale host or microhabitat covariates. As a result, some compartment-level, microhabitat, or host-related heterogeneity may not have been fully resolved. Future studies with expanded sampling should incorporate host traits and environmental variables more explicitly and combine metatranscriptomics, metabolomics, targeted isolation, and co-culture experiments to link taxonomic successions with functional processes and test the hypothesized interactions among key taxa (20).

## MATERIALS AND METHODS

### Sample collection and processing

Samples were collected in August 2023 from the Sejila Mountains in Nyingchi City, Tibet Autonomous Region, China. Four sampling sites were established within the same mountain forest system, with coordinates ranging from 94.704942°E, 29.634998°N to 94.725479°E, 29.648194°N and elevations from 3,700 to 4,300 m. Pairwise distances among sites ranged from approximately 0.53 to 2.47 km. Although geographically separated, all sites belonged to the same regional forest landscape and shared broadly similar environmental conditions. A total of 14 trees were sampled, including 5 healthy, 4 asymptomatic, and 5 symptomatic trees (Table 1). These health categories were represented across all four sampling sites. Detailed metadata for all samples, including sampling site, geographic coordinates, elevation, health status, and tissue compartment, are provided in Table S5. During field selection, trees of broadly similar size and overall appearance were preferentially chosen, and individuals with obvious non-disease-related mechanical damage or extreme growth form were avoided. Tree health status was determined through a combination of external visual inspection and internal assessment of increment core samples. External visual inspection focused on visible trunk symptoms potentially associated with heart rot, particularly the presence of fruiting bodies, external discoloration, and other obvious stem abnormalities. Healthy trees showed no visible external symptoms or internal abnormalities; asymptomatic trees showed no obvious external symptoms but had internal heartwood decay; and symptomatic trees bore fruiting bodies and showed advanced internal decay. Core samples were obtained at breast height using a hand-operated increment borer disinfected with 75% ethanol before each use. The material used for downstream analyses was taken from the increment core itself; loose sawdust generated during boring was not collected as a separate sample. For each tree, two tissue compartments were sampled from the increment core: an outer section located approximately 2–3 cm from the bark (near-bark compartment) and an inner section located approximately 2–3 cm from the pith (near-pith compartment). This predefined positional sampling scheme was used to standardize sampling depth across trees. Samples were immediately transferred into sterile centrifuge tubes, flash-frozen in liquid nitrogen, and stored at −80°C until DNA extraction.

### DNA extraction and amplicon sequencing

Total genomic DNA was extracted from approximately 0.5 g of the core sample using the MOBIO PowerSoil DNA Isolation Kit (MOBIO, USA) (32). DNA concentration and purity were determined using a NanoDrop 2000 spectrophotometer (Thermo Fisher Scientific, USA). The V5–V7 hypervariable region of the bacterial 16S rRNA gene was amplified using the primers 799F (5′-AACMGGATTAGATACCCKG-3′) and 1193R (5′-ACGTCATCCCCACCTTCC-3′) (33). The fungal ITS1 region was amplified using primers ITS1F (5′-CTTGGTCATTTAGAGGAAGTAA-3′) and ITS2 (5′-GCTGCGTTCTTCATCGATGC-3′) (34). The PCR mixture (50 μL) contained 25 μL of 2× Premix Taq (TaKaRa, China), 1 μL each of forward and reverse primers (10 μM), and 50 ng of DNA template and was brought to volume with nuclease-free water. PCR was performed on a Biometro TONE 96G thermocycler (Jena, Germany) using the following program: initial denaturation at 94°C for 5 min; 30 cycles of 94°C for 30 s, 52°C for 30 s, and 72°C for 30 s; and a final extension at 72°C for 10 min.

The PCR products were analyzed using 1.5% agarose gel electrophoresis. Qualified products were purified using the E.Z.N.A. Gel Extraction Kit (Omega, USA), and sequencing libraries were constructed according to the ALFA-SEQ DNA Library Prep’s instructions. Library quality was assessed for fragment size distribution using Qsep400 (BiOptic, China) and quantified precisely using Qubit 4.0 (Thermo Fisher Scientific, USA). Finally, paired-end 250-bp sequencing was performed on an Illumina HiSeq 2500 platform (Majorbio, China) (35).

### Sequencing data processing and analysis

Raw sequencing data were subjected to quality control using fastp (v0.14.1), and primer sequences were removed using Cutadapt to obtain clean reads (36, 37). Paired-end clean reads were merged into raw tags using the fastq_mergepairs command in USEARCH (v10) (parameters: minimum overlap length 16 bp and maximum mismatches 5 bp) (38). Quality filtering was performed again using fastp to obtain the final clean tags for analysis. The DADA2 algorithm within the QIIME2 environment (version 2020.6) was used for denoising to generate amplicon sequence variants (39, 40). Bacterial 16S rRNA gene amplicon sequence variants (ASVs) were taxonomically annotated using the SILVA database (v138), and fungal ITS ASVs were annotated using the UNITE database (v8.0) (confidence threshold set to 0.8) (41, 42). After annotation, ASVs identified as chloroplasts, mitochondria (for bacteria), or of plant origin (for fungi) were removed. Across the 28 samples included in this study, chloroplast-derived reads in the bacterial 16S data set accounted for 0.03%–36.85% of raw reads (mean = 7.73%), whereas mitochondria-derived reads accounted for 0%–0.09% (mean = 0.022%). Chloroplast- and mitochondria-derived reads were not detected in the fungal ITS data set. ASVs with a total sequence count of less than two were also filtered out, resulting in the final ASV abundance table.

### Community diversity analysis

For alpha diversity analyses, bacterial samples were rarefied to 14,646 sequences per sample, and fungal samples were rarefied to 45,525 sequences per sample. Alpha diversity indices (Shannon indices) for the bacterial and fungal communities were calculated using QIIME2 (40). The significance of intergroup differences in alpha diversity was assessed using the Kruskal–Wallis test followed by *post hoc* pairwise comparisons (Wilcoxon rank-sum test with Bonferroni correction).

Beta diversity was visualized via PCoA based on the Bray–Curtis distance matrices. To assess the dispersion of samples within each group, 0.95 confidence level ellipses based on a multivariate *t* distribution were drawn for each group on the PCoA plots using the ggplot2 package (43). PERMANOVA was performed using the adonis function in the vegan package (v2.5-7) with 999 permutations to test the effects of disease status, tissue compartment, and their interaction on community structure (44). PERMANOVA results are reported as *R*^2^, pseudo-*F*, and *P* values, where pseudo-*F* is the permutation-based test statistic analogous to the *F* statistic in conventional ANOVA. In addition, to evaluate whether the sampling site affected community structure, a separate site-based PERMANOVA was performed using the four sampling sites as grouping factors for both bacterial and fungal communities. *Post hoc* pairwise comparisons for disease severity were performed, and *P* values were adjusted using the Bonferroni method.

### Community composition and differential abundance analysis

The average relative abundance within each group was calculated at the class and family level. To highlight the dominant taxa, we displayed only taxa with a relative abundance ≥1% that were not unclassified sequences, selecting the top 15 dominant classes and top 30 dominant families to create stacked bar charts.

To identify key microbial taxa associated with the disease status, we performed differential analyses at two levels. First, the linear discriminant analysis effect size algorithm was used to systematically screen for biomarkers across multiple taxonomic ranks that differed significantly among the healthy, asymptomatic, and symptomatic groups (LDA score threshold set to 3.5) (32). Second, to focus on relatively abundant families while avoiding the exclusion of biologically informative taxa below the 1% display threshold, we performed pairwise group difference analyses of the top 20 most abundant bacterial and fungal families with a mean relative abundance of at least 0.1%. We used the nonparametric Wilcoxon rank-sum test for pairwise comparisons, with the significance level set at 0.05. *P* values were adjusted for multiple testing using the Benjamini-Hochberg (FDR) method (45). Confidence intervals were calculated using a bootstrap procedure with a 95% confidence level.

### Co-occurrence network analysis

To investigate microbial interaction patterns under different health states, we constructed intrakingdom (bacteria-bacteria and fungi-fungi) and cross-kingdom (bacteria-fungi) co-occurrence networks separately for healthy, asymptomatic, and symptomatic groups at the ASV level. The bacterial and fungal ASV abundance tables were merged for data pre-processing. For intrakingdom networks, ASVs present in less than 20% of the samples within each group were removed, and all remaining ASVs were used for network construction. For the cross-kingdom networks, to reduce noise and control network complexity while focusing on core community members, we applied a more stringent filtering step: ASVs detected in fewer than 30% of samples were removed, and only the top 100 bacterial ASVs and the top 100 fungal ASVs by mean relative abundance in each group were retained for subsequent analysis. Based on the filtered ASV relative abundance data, the Spearman’s rank correlation coefficients (*ρ*) between all ASV pairs were calculated using the Hmisc R package (v4.7.0) (46). To control the false-positive risk arising from large-scale pairwise comparisons, all calculated *P* values were subjected to Benjamini-Hochberg (FDR) correction (45). Ultimately, thresholds of |Spearman’s *ρ*| ≥ 0.6 and FDR-adjusted *P* value (*q* value) < 0.05 were set to define statistically robust microbial associations, which were defined as edges in the network. The nodes represent individual ASVs, resulting in an undirected network.

The topological properties of each network, including the number of nodes, number of edges, network density, average degree, proportion of positive/negative edges, average path length, and modularity index, were calculated using the igraph R package (v1.5.1) (47). To further quantify the differences in these topological properties between groups, the following statistical tests were performed: (i) chi-square tests were used to compare the proportions of positive/negative edges and the distribution of bacterial/fungal nodes across disease groups; (ii) permutation tests (999 permutations) were used to compare network density across groups. Finally, network visualization was performed using Gephi software (v0.10.1) (48).

## Data Availability

Raw sequencing data have been deposited in the NCBI Sequence Read Archive under BioProject ID PRJNA1377932; other data are provided in the article and its supplemental materials.

## SUPPLEMENTAL MATERIAL

Figure S1 spectrum.03904-25-s0001.docx Per-sample family-level composition of bacterial and fungal communities across all samples.

Table S1 spectrum.03904-25-s0002.xlsx Fungal relative abundance at the class and family levels.

Table S2 spectrum.03904-25-s0003.xlsx Fungal differential abundance analysis (*P* values) at the phylum, class, and genus levels.

Table S3 spectrum.03904-25-s0004.xlsx Bacterial differential abundance analysis (*P* values) at the phylum, class, and genus levels.

Table S4 spectrum.03904-25-s0005.xlsx Bacterial relative abundance at the class and family levels.

Table S5 spectrum.03904-25-s0006.docx Metadata for all samples, including sampling site, geographic coordinates, elevation, health status, and tissue compartment.

ASM does not own the copyrights to Supplemental Material that may be linked to, or accessed through, an article. The authors have granted ASM a non-exclusive, world-wide license to publish the Supplemental Material files. Please contact the corresponding author directly for reuse.
