# Supplementary material for: Structures of the endophytic microbiota during heart rot development in Abies georgei var. smithii
Source: Microbiol Spectr. 2026 Jun 23;14(8):e03904-25. doi: 10.1128/spectrum.03904-25 (PMC13436395; doi:10.1128/spectrum.03904-25)
Supplement: Supplementary file 1 [file spectrum.03904-25-s0001.docx]

**Fig. S1.** Per-sample family-level composition of bacterial and fungal communities across all samples. (A) Bacterial family composition of individual samples. (B) Fungal family composition of individual samples. Samples are grouped by disease status as healthy (H), asymptomatic (AS), and symptomatic (S), and within each group are ordered by tissue compartment (bark and pith).
