# Supplementary material for: Structures of the endophytic microbiota during heart rot development in Abies georgei var. smithii
Source: Microbiol Spectr. 2026 Jun 23;14(8):e03904-25. doi: 10.1128/spectrum.03904-25 (PMC13436395; doi:10.1128/spectrum.03904-25)
Supplement: Supplementary file 2 [file spectrum.03904-25-s0006.docx]

**Table S5.** Metadata for all wood-core samples used in this study, including sampling site, geographic coordinates, elevation, health status, and tissue compartment.

| Sample ID | Tree ID | Site | Longitude | Latitude | Elevation (m) | Health status | Compartment |
| --- | --- | --- | --- | --- | --- | --- | --- |
| H1_bark | H1 | 1 | 94.725479 | 29.648194 | 3700 | Healthy | Near bark |
| H1_pith | H1 | 1 | 94.725479 | 29.648194 | 3700 | Healthy | Near pith |
| H2_bark | H2 | 1 | 94.725479 | 29.648194 | 3700 | Healthy | Near bark |
| H2_pith | H2 | 1 | 94.725479 | 29.648194 | 3700 | Healthy | Near pith |
| AS1_bark | A1 | 1 | 94.725479 | 29.648194 | 3700 | Asymptomatic | Near bark |
| AS1_pith | A1 | 1 | 94.725479 | 29.648194 | 3700 | Asymptomatic | Near pith |
| S1_bark | S1 | 1 | 94.725479 | 29.648194 | 3700 | Symptomatic | Near bark |
| S1_pith | S1 | 1 | 94.725479 | 29.648194 | 3700 | Symptomatic | Near pith |
| H3_bark | H3 | 2 | 94.710361 | 29.643917 | 3900 | Healthy | Near bark |
| H3_pith | H3 | 2 | 94.710361 | 29.643917 | 3900 | Healthy | Near pith |
| AS2_bark | A2 | 2 | 94.710361 | 29.643917 | 3900 | Asymptomatic | Near bark |
| AS2_pith | A2 | 2 | 94.710361 | 29.643917 | 3900 | Asymptomatic | Near pith |
| S2_bark | S2 | 2 | 94.710361 | 29.643917 | 3900 | Symptomatic | Near bark |
| S2_pith | S2 | 2 | 94.710361 | 29.643917 | 3900 | Symptomatic | Near pith |
| H4_bark | H4 | 3 | 94.707999 | 29.638952 | 4100 | Healthy | Near bark |
| H4_pith | H4 | 3 | 94.707999 | 29.638952 | 4100 | Healthy | Near pith |
| AS3_bark | A3 | 3 | 94.707999 | 29.638952 | 4100 | Asymptomatic | Near bark |
| AS3_pith | A3 | 3 | 94.707999 | 29.638952 | 4100 | Asymptomatic | Near pith |
| S3_bark | S3 | 3 | 94.707999 | 29.638952 | 4100 | Symptomatic | Near bark |
| S3_pith | S3 | 3 | 94.707999 | 29.638952 | 4100 | Symptomatic | Near pith |
| S4_bark | S4 | 3 | 94.707999 | 29.638952 | 4100 | Symptomatic | Near bark |
| S4_pith | S4 | 3 | 94.707999 | 29.638952 | 4100 | Symptomatic | Near pith |
| H5_bark | H5 | 4 | 94.704942 | 29.634998 | 4300 | Healthy | Near bark |
| H5_pith | H5 | 4 | 94.704942 | 29.634998 | 4300 | Healthy | Near pith |
| AS4_bark | A4 | 4 | 94.704942 | 29.634998 | 4300 | Asymptomatic | Near bark |
| AS4_pith | A4 | 4 | 94.704942 | 29.634998 | 4300 | Asymptomatic | Near pith |
| S5_bark | S5 | 4 | 94.704942 | 29.634998 | 4300 | Symptomatic | Near bark |
| S5_pith | S5 | 4 | 94.704942 | 29.634998 | 4300 | Symptomatic | Near pith |
